# Sarcopenia and osteosarcopenia among patients undergoing hemodialysis

**DOI:** 10.3389/fendo.2023.1181139

**Published:** 2023-05-17

**Authors:** Ting Xiang, Ping Fu, Li Zhou

**Affiliations:** Division of Nephrology, Kidney Research Institute, West China Hospital of Sichuan University, Chengdu, Sichuan, China

**Keywords:** osteosarcopenia, Sarcopenia, osteoporosis, mortality, hemodialysis

## Abstract

**Background:**

Sarcopenia and osteoporosis are closely interconnected and associated with adverse health outcomes. Osteosarcopenia is the concurrent presence of the two conditions and has rarely been reported in hemodialysis patients. Whether hemodialysis patients with osteosarcopenia are at greater risk of mortality than those with either condition alone remains unknown. The aim of this study was to explore the prevalence of sarcopenia and its association with osteoporosis and to determine its impact on survival risk in hemodialysis patients.

**Methods:**

A total of 209 adults undergoing hemodialysis were enrolled from the dialysis center in the West China Hospital of Sichuan University, and our study was registered at the Chinese Clinical Trial Register (number: ChiCTR2100043932). Muscle mass, handgrip strength, bone mineral density (BMD), and biochemical parameters were assessed. All deaths were recorded during a follow-up of 35.15 ± 15.37 months.

**Results:**

Seventy-eight patients were diagnosed with sarcopenia, with a prevalence of 37.3%. After adjustment for potential confounders, age (OR=1.094, P <0.001), female sex (OR= 3.44, P =0.005), diabetes (OR=3.756, P =0.008), CRP (OR=1.09, P =0.015), serum magnesium (OR=0.755, p=0.042) and BMI (OR=0.701, P <0.001) were independently associated with sarcopenia. Among the 209 patients, 103 patients completed the BMD assessment. The prevalence of osteosarcopenia was 22.3%, while 20.4% of participants had sarcopenia alone and 12.6% had osteoporosis alone. The proportions of patients who died were 13.0% for nonsarcopenia&nonosteoporosis, 15.4% for osteoporosis alone, 47.6% for sarcopenia alone, and 52.2% for osteosarcopenia. Cox regression analysis showed that osteosarcopenia was independently associated with all-cause mortality (HR=3.74, 95% CI: 1.172-11.938), while osteoporosis alone and sarcopenia alone were not.

**Conclusion:**

Patients undergoing hemodialysis had a high incidence of sarcopenia and osteosarcopenia, muscle mass and strength showed a significant association with BMD, and osteosarcopenia might have a powerful impact on mortality in those patients.

**Clinical trial registration:**

http://www.chictr.org.cn/, identifier ChiCTR2100043932.

## Introduction

Sarcopenia (SP) refers to the gradual decline in both skeletal muscle function and mass and was first proposed in the 1980s (1). It is a progressive and systemic skeletal muscle disorder and has been reported to be associated with adverse clinical outcomes such as physical disability, falls,and all-cause mortality (2–5). Ageing, low nutrient intake, low activity and sedentary lifestyle are underlying causes of sarcopenia (6). There are multiple definitions of sarcopenia, including the European Working Group on Sarcopenia in Older People (EWGSOP), International Working Group on Sarcopenia (IWGS) and Asian Working Group for Sarcopenia (AWGS). All definitions are based on muscle mass, muscle strength, and physical performance but different cut-off points, so there is a lack of standard definitions in clinical practice (7). Sarcopenia occurs commonly in older people and is defined as an age-related disease. While it has also been found in other diseases, such as some cancers, endocrine diseases and metabolic disorders, disease-related sarcopenia is currently included in many research studies (8, 9). End-stage renal disease (ESRD) is a global health concern that has attracted increasing attention. Dialysis, as the main renal replacement therapy, accounts for 62.7% of ESRD patients, and the related complications are also gradually increasing (10). The accelerated process of protein wasting, multiple metabolic derangements, and nutrient deficiency may induce accelerated degradation of muscle mass and lead to sarcopenia in ESRD patients undergoing maintenance hemodialysis (MHD) (11, 12). Studies have reported that more than 20% of ESRD patients have sarcopenia, which is significantly higher than the prevalence in the general population (13, 14).

Muscle synthesis and bone metabolism seem to be interconnected. Skeletal muscle can secrete factors to regulate bone metabolism, such as myostatin, IGF‐1, and FGF-2. Bone also functions as an endocrine organ to produce cytokines that act on muscle, including FGF23, sclerostin, and osteocalcin (15). Osteoporosis (OP) is a disease characterized by decreased bone mineral density (BMD) and damaged bone structure, leading to a risk of fractures (16). Skeletal muscle loss often coincides with low bone density, and the prevalence of osteoporosis among patients with sarcopenia is higher than that among those without sarcopenia. Osteoporosis is also an independent predictor of sarcopenia (17).

Osteosarcopenia (OS) is defined by the concurrent presence of osteoporosis and sarcopenia, a new concept proposed in 2009 by Duque and colleagues (18). It is worth noting that osteosarcopenia is a unique disease that involves the combination of low bone density and muscle mass, strength, and/or function. Currently, the criteria for osteosarcopenia are inconsistent, with some studies referring to osteopenia and sarcopenia, while others are defined as osteoporosis and sarcopenia (19). It has been reported that osteoporosis and sarcopenia share common risk factors, so osteosarcopenia is associated with aging, low nutritional status, low physical function and some chronic diseases (20). The coexistence of low bone mass and the low muscle mass, strength and function will contribute to a worse outcome than each one alone (21). The concurrent presence of osteoporosis and sarcopenia can affect each other and lead to a worsening of outcomes, such as higher risk of falls, fractures, and mortality (22, 23). Inoue et al. reported that the incidence of social frailty was 8.0% in robust patients, 11.8% in osteoporosis alone, 17.9% in sarcopenia alone, and 29.1% in osteosarcopenia (24). In addition, a study reported that the fracture risk is 3.5-fold higher than that in sarcopenia and osteoporosis alone (25).

The prevalence of bone metabolism disorders and aggravated muscle wasting in ESRD patients leads to a high incidence of osteosarcopenia. However, few studies have evaluated the association between sarcopenia and osteoporosis in MHD patients. And osteosarcopenia as a new concept, there are lacking of studies to explore its effect on clinical outcomes. Early detection of osteosarcopenia in MHD patients may improve prognosis and reduce mortality. Our study aimed to investigate the actual situation of sarcopenia and the relationship between sarcopenia and osteoporosis in MHD patients.

## Materials and methods

### Subjects

**Figure 1:**
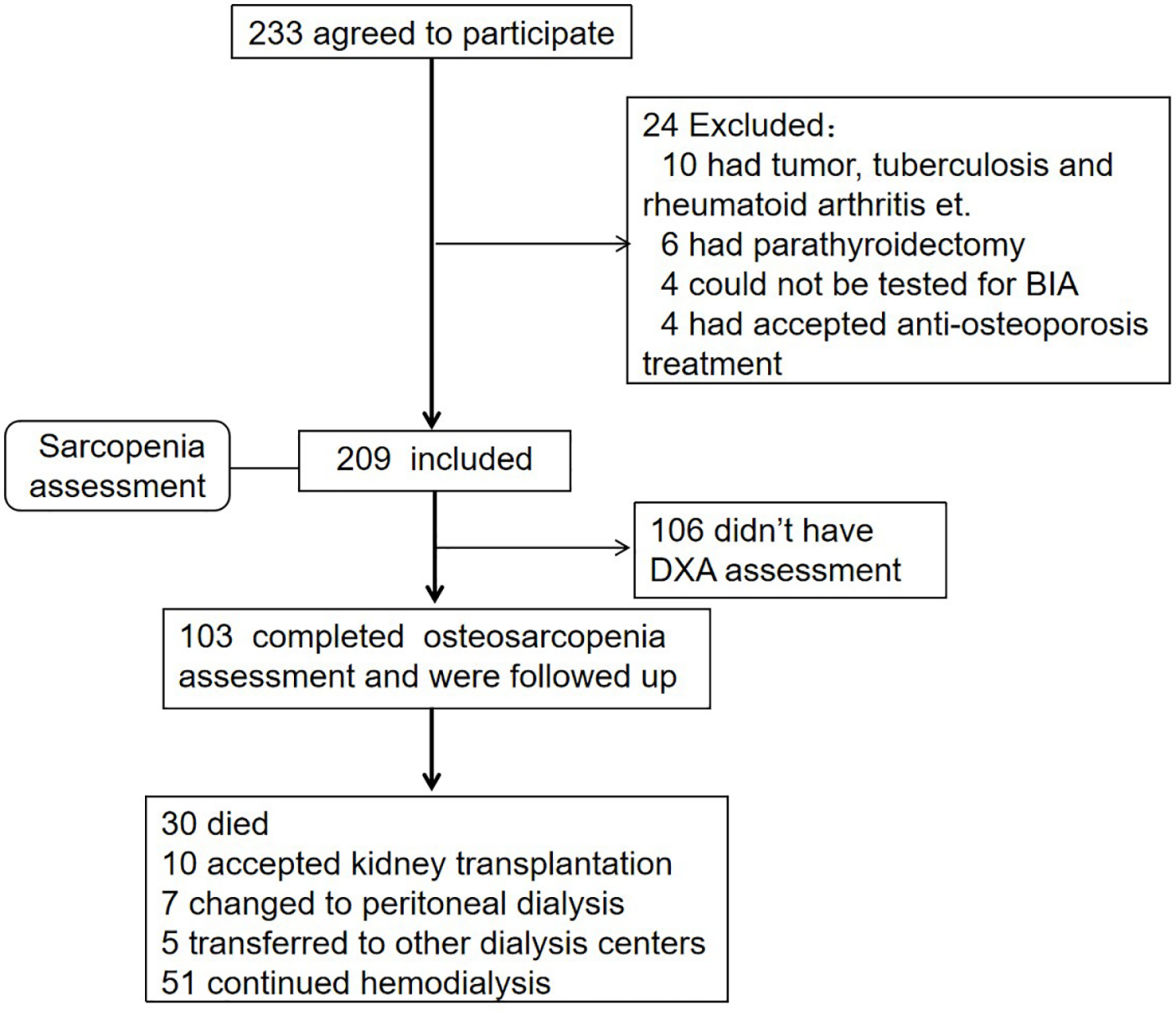
Flow diagram. A total of 209 participants had accepted sarcopenia assessment, and 103 completed the DXA test concurrently and were followed up for 35.15 ± 15.37 months.

This prospective study was conducted in MHD patients from the dialysis center in the West China Hospital of Sichuan University between July 2018 and March 2020 (shown in **Figure 1**). The inclusion criteria were as follows (1): being under MHD for more than 3 months (2); at least 18 years of age; and (3) consent to participate in this study. The exclusion criteria were as follows (1): accepted anti-osteoporosis treatment in the past 6 months, such as bisphosphonates (2); patients for which BIA could not be performed (such as in patients who underwent pacemaker installation and amputation surgery); and (3) had other diseases affecting bone metabolism.

Baseline data were collected between July 2018 and March 2020. The follow-up deadline was October 1, 2022. The endpoint was overall mortality, and censored events included transfer to another dialysis center, kidney transplantation, conversion to peritoneal dialysis, and survival at the deadline. Our study was registered at the Chinese Clinical Trial Register (http://www.chictr.org.cn/; ChiCTR2100043932), and was approved by the Ethics Committee of West China Hospital of Sichuan University (2020-446).

### General data collection

General information was collected by medical records or questionnaires as follows: age, body mass index (BMI), dialysis vintage, previous osteoporosis fracture and other underlying conditions.

### Laboratory biochemical index

Avoiding the influence of dialysate on results, blood samples were obtained before performing hemodialysis, and laboratory indicators included albumin, calcium, magnesium, phosphorus, parathyroid hormone (PTH), alkaline phosphatase (ALP), 25-hydroxyvitamin D [25(OH)D], and C-reactive protein (CRP). Serum calcium was adjusted by the equation: measured calcium (mmol/l) + 0.02x [40-serum albumin (g/dL)].

### Anthropometric measurements

Body composition was estimated by the BIA method (InbodyS720, Biospace, Seoul, South Korea). The appendicular skeletal muscle mass index (ASMI) was calculated using the following formula: muscle mass of the four limbs (kg)/height^2^ (m^2^). Handgrip strength (HGS) was assessed on the non-fistula side using a digital grip strength dynamometer (CAMRY, EH101), three measurements were averaged and used in the analyses. Total hip, femoral neck, and lumbar spine BMD (g/cm2) measurements were performed using dual-energy X-ray absorptiometry (DXA) (GE Lunar, ME + 212243, USA).

### Diagnosis of sarcopenia, osteoporosis and osteosarcopenia

Sarcopenia was defined as the loss of skeletal muscle mass and strength according to the AWGS criteria. The cut-off points of the ASMI were 7.0kg/m^2^ for males and 5.4 kg/m^2^ for females, and 26 kg for males and 18 kg for females for handgrip strength. Osteoporosis was diagnosed as T≤-2.5 measured by DXA, according to the World Health Organization (WHO) criteria. Osteosarcopenia is defined as the presence of sarcopenia combined with osteoporosis.

### Statistical analyses

Continuous variables were presented as the mean ± SD or median and interquartile interval according to the distribution and percentages for categorical variables. The comparison of quantitative variables was performed using the T test or Mann−Whitney U test (two groups) and one-way ANOVA or Kruskal−Wallis test (three or more groups). Chi-square tests were used for qualitative variables. Logistic regression (Forwards: LR) was used to explore the independent factors associated with sarcopenia. Pearson’s linear analysis was used to test the correlation between ASMI, HGS and BMD. Cox regression proportional hazards models were used to estimate the adjusted risk of mortality, after adjustment for age, sex, dialysis vintage, diabetes, cardiovascular disease, and fracture history et. P < 0.05 was considered statistically significant. Analyses were performed by SPSS Statistics 23 (IBM, Armonk, NY).

## Result

### Patient demographics

The studied sample comprised 209 patients (47.4% male), with a mean age of 58.45 ± 15.31 years (range, 21–88). In all, 78 (37.3%) participants presented sarcopenia. The characteristics of the patients are described in **Table 1**. Sarcopenia patients, in this study, were more likely to be female (p=0.01) and older (P <0.001), and had a lower BMI (P =0.006) than nonsarcopenia patients. Regarding the biochemical parameters, the sarcopenia group had higher CRP levels (P <0.001) and lower serum phosphorus (P =0.033), serum magnesium (P=0.004), and albumin (P=0.002) levels. The prevalence rates of fracture history (P =0.002) and diabetes (P =0.006) were higher in the sarcopenia group than that in the nonsarcopenia group.

**Table 1:** Demographic and clinical characteristics of participants.

	sarcopenia (n=78)	nonsarcopenia (n=131)	p
Age(years)	66.92 ± 13.64	53.42 ± 14.01	<0.001^*^
Female, n (%)	50(64.1%)	60(45.8%)	0.01 ^*^
Dialysis vintage(years)	5(3,10)	5(2, 8)	0.059
ASMI (kg/m^2^)	5.39 ± 0.91	6.84 ± 1.19	<0.001^*^
HGS (kg)	13.4 ± 5.14	24.17 ± 8.89	<0.001^*^
BMI (kg/m^2^)	21.26 ± 2.82	22.79 ± 3.35	<0.001^*^
Calcium(mmol/L)	2.32 ± 0.27	2.28 ± 0.23	0.203
Phosphorus(mmol/L)	1.76 ± 0.55	1.91 ± 0.46	0.033 ^*^
PTH (pmol/L)	31.78 (13.56, 55.99)	33.68 (19.56, 52.56)	0.329
Magnesium (mmol/L)	1.03 ± 0.14	1.1 ± 0.19	0.004^*^
ALP (IU/L)	100.5(73,135.25)	82(65,101)	0.21
25(OH)D (nmol/L)	50.3 ± 22.23	53.39 ± 22.74	0.357
CRP(mg/L)	5.22(2.85,8.93)	3.22(1.97,5.65)	<0.001^*^
Albumin (g/L)	40.41 ± 4.58	42.29 ± 4.01	0.002^*^
Fracture history, n (%)	16(20.5%)	8(6.1%)	0.002^*^
Diabetes, n (%)	29(37.2%)	26(19.5%)	0.006^*^
Coronary artery disease, n (%)	19(24.4%)	20(15.3%)	0.103

ASMI, appendicular skeletal muscle mass index; HGS, handgrip strength; BMI, body mass index; PTH, parathyroid hormone; ALP, alkaline phosphatase; 25(OH)D, 25-hydroxyvitamin D; CRP, C-reactive protein *p<0.05

### Factors associated with sarcopenia

The above variables with P<0.1 were included in the multivariate logistical regression analysis. Age (OR=1.094, P <0.001), female sex (OR= 3.44, P =0.005), diabetes (OR=3.756, P =0.008), and CRP (OR=1.09, P =0.015) were independent risk factors for sarcopenia, and serum magnesium (OR=0.755, p=0.042) and BMI (OR=0.701, P <0.001) were protective factors for sarcopenia in our study, as shown in **Table 2**.

**Table 2:** Factors associated with sarcopenia.

	Logistic regression		
	OR	95%CI P	
Age (per one year increase)	1.094	1.061-1.127	<0.001^*^
Female	3.44	1.438-8.227	0.005^*^
BMI (per one kg/m^2^ increase)	0.701	0.596-0.823	<0.001^*^
CRP (per one mg/L increase)	1.09	1.017-1.169	0.015^*^
Magnesium (per 0.1 mmol/L increase)	0.755	0.725-0.989	0.042^*^
Phosphorus (per one mmol/L increase)	–	–	0.656
Albumin (per one g/L increase)	–	–	0.987
Fracture history	–	–	0.251
Diabetes	3.756	1.416-9.963	0.008^*^
Dialysis vintage (per one year increase)	–	–	0.5

BMI, Body mass index; CRP, C-reactive protein.

*p<0.05.

### The prevalence of osteosarcopenia

Among the 209 patients, 103 patients completed the BMD assessment. According to the results, patients were divided into nonsarcopenia&nonosteoporosis (44.7%), osteoporosis alone (12.6%), sarcopenia alone (20.4%), and osteosarcopenia (22.3%). The characteristics of patients according to osteosarcopenia categories are described in **Table 3**. Osteosarcopenia patients had significantly higher rates of fracture history and mortality and a lower albumin level. Sarcopenia alone patients were older and had higher CRP levels and rates of diabetes. The 25(OH)D levels were significantly lower in osteoporosis alone patients.

**Table 3:** Prevalence of patient classification based on osteosarcopenia.

	NONS n=46	OP alone n=13	SP alone n=21	OS n=23
Age (year)	52.04 ± 15.24	60.7 ± 17.76	68.2 ± 14.59^*^	66.82 ± 10.64
Female, n (%)	24 (52.2%)	12 (92.3%)^‡^	9 (42.9%)†^§^	19 (82.6%)^‡^
Dialysis vintage (years)	5 (3,6)	5 (2.5,8.5)	8 (4.2,10)	4 (3,8)
BMD (g/cm^2^)				
lumbar spine	1.07 ± 0.17†^§^	0.81 ± 0.15^*‡^	1.14 ± 0.3†^§^	0.84 ± 0.14^*‡^
femoral neck	0.83 ± 0.12†^§^	0.61 ± 0.07^*‡^	0.78 ± 0.11†^§^	0.64 ± 0.1^*‡^
total hip	0.88 ± 0.13†^§^	0.64 ± 0.08 ^*‡^	0.85 ± 0.13†^§^	0.68 ± 0.11^*‡^
BMI (kg/m^2^)	22.85 ± 3.23	23.65 ± 4.41^§^	22.6 ± 3.34	21.25 ± 2.67^†^
ASMI (kg/m^2^)	6.77 ± 1.21^‡^	6.15 ± 0.76^§^	5.71 ± 0.85^*^	5.08 ± 0.92^*†^
HGS (kg)	22.91 ± 8.77^‡^	19.14 ± 5.73^§^	15.07 ± 5.04^*^	12.34 ± 4.97^*†^
Calcium (mmol/L)	2.45 ± 0.25	2.34 ± 0.25	2.36 ± 0.21	2.35 ± 0.22
Magnesium (mmol/L)	1.07 ± 0.15	1.12 ± 0.14	1.01 ± 0.15	1.05 ± 0.14
Phosphorus (mmol/L)	1.82 ± 0.39	1.93 ± 0.46^‡^	1.79 ± 0.59^†^	1.87 ± 0.52
25 (OH)D (nmol/L)	53.79 ± 20.49^†^	37.85 ± 9.81^*^	49.16 ± 18.72	49.57 ± 20.6
PTH (pmol/L)	28.89 (15.19,56.76)	18.36 (13.62,37.3)	27.07 (7.95,59.41)	22.56 (11.42,43.29)
ALP (IU/L)	79 (58.5,95.5)	77 (63,108.5)	99 (75,133.5)	77 (62,115)
CRP (mg/L)	4.79 (2.48,7.28)	3.18^‡^ (1.37,6.84)	5.71^†§^ (4.18,18.55)	3.13^‡^ (2.21,6.23)
Albumin (g/L)	42.51 ± 4.46^§^	41.89 ± 3.91	40.82 ± 6.32	40.93 ± 3.51^*^
Diabetes, n (%)	11 (24.4%)	1 (7.7%)^‡^	11 (52.4%)^†^	8 (34.8%)
Cardiovascular disease, n (%)	6 (13.1%)	2 (15.4%)	8 (38.1%)	3 (13%)
Fracture history, n (%)	2 (4.3%)†^§^	4 (30.8%) ^*^	5 (23.8%)	9 (39.1%) ^*^
Mortality, n (%)	6 (13.0%)‡^§^	2 (15.4%)	10 (47.6%) ^*^	12 (52.2%) ^*^

*, significant difference from normal; †, significant difference from osteoporosis only; ‡, significant difference from sarcopenia only; §, significant difference from osteosarcopenia.

NONS, nonosteoporosis&nonsarcopenia; OP, osteoporosis; SP, sarcopenia; OS, osteosarcopenia; BMD, bone mineral density; ASMI, appendicular skeletal muscle mass index; HGS, handgrip strength; BMI, body mass index; PTH, parathyroid hormone; ALP, alkaline phosphatase; 25 (OH)D, 25-hydroxyvitamin D; CRP, C-reactive protein.

### Correlation between sarcopenia and osteoporosi

**Figure 2:**
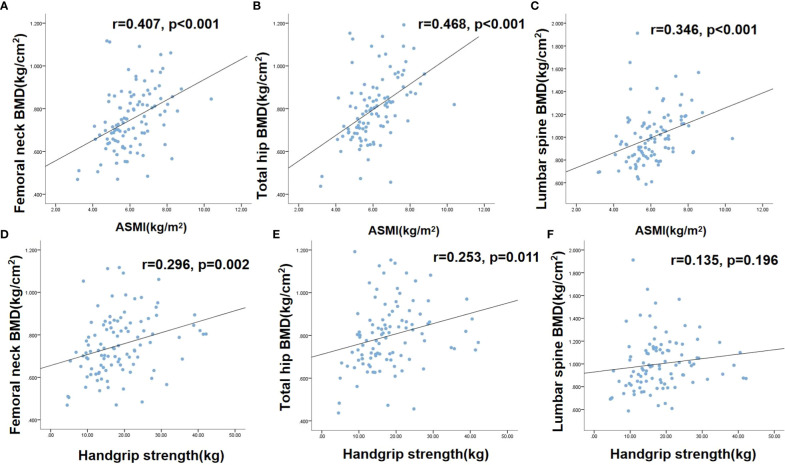
The correlations between sarcopenia and osteoporosis **Figure 1**. **(A)** correlation between femoral neck BMD and ASMI; **(B)** correlation between total hip BMD and ASMI; **(C)** correlation between lumbar spine BMD and ASMI; **(D)** correlation between femoral neck BMD and handgrip strength; **(E)** correlation between total hip BMD and handgrip strength. **(F)** correlation between lumbar spine BMD and handgrip strength. BMD, Bone mineral density; ASMI, Appendicular skeletal muscle mass index.

As described above, 63.9% (23/36) of patients with osteoporosis had sarcopenia in this cohort, and the risk of sarcopenia was 3.875-fold higher in patients with osteoporosis than in those without osteoporosis. There was a significant, positive correlation between ASMI and BMD of the lumbar spine (r = 0.346), femoral neck (r = 0.407), and total hip (r = 0.468) (P < 0.001 for all). Handgrip strength was significantly correlated with the BMD of the femoral neck (r = 0.296, P = 0.002) and total hip (r = 0.253, P = 0.011) but not with the BMD of the lumbar spine, as shown in **Figure 2**.

### Postoperative survival analysis

The respective proportions of patients in this study who died over a mean follow-up period of 35.15 ± 15.37 months were 13.0% for nonsarcopenia&nonosteoporosis, 15.4% for osteoporosis alone, 47.6% for sarcopenia alone, and 52.2% for osteosarcopenia. Cox proportional regression analysis showed that osteosarcopenia and sarcopenia alone had an increased hazard for mortality in unadjusted models. After adjustment for age and sex, the increase hazard was disappeared in sarcopenia alone, while remained significant for osteosarcopenia patients (HR=3.74, 95% CI: 1.172-11.938), even further adjusted dialysis vintage, diabetes, cardiovascular disease, and fracture history et. (shown in **Table 4**)

**Table 4:** Cox regression models for mortality according to osteosarcopenia.

Model		COX regression analysis	
		HR	P
1	OP alone	1.12 (0.225-5.576)	0.890
	SP alone	3.146 (1.132-8.742)	0.028^*^
	OS	4.345 (1.626-11.609)	0.003^*^
2	OP alone	0.911 (0.162-5.131)	0.916
	SP alone	1.987 (0.618-6.394)	0.249
	OS	3.63 (1.256-10.486)	0.017^*^
3	OP alone	0.841 (0.135-5.233)	0.853
	SP alone	0.356 (0.527-5.951)	0.356
	OS	3.74 (1.172-11.938)	0.026^*^

Model 1, unadjusted.

Model 2, adjusted for age, sex.

Model 3, adjusted Model 2+ dialysis vintage, diabetes, cardiovascular disease, fracture history.

OP, osteoporosis; SP, sarcopenia; OS, osteosarcopenia.

*p<0.05

## Discussion

The occurrence of sarcopenia in ESRD have attracted the worldwide attention nowadays (26, 27). Due to the different diagnostic criteria, there was great variability in the prevalence of sarcopenia in hemodialysis patients, approximately 13.7%-73.5% (28). A standardized diagnostic method for sarcopenia is important for clinical diagnosis and research in the future. According to the AWGS criteria, sarcopenia was diagnosed in 37.3% of the patients in our study, and 28% for male 50% for female respectively. Sex hormones on muscle function have been studied, different effects of androgens and estrogens lead to differences in skeletal muscle morphology and function. Testosterone is widely believed to affect muscle protein synthesis and muscular regeneration to increase muscle mass and strength (29, 30). The effect of estrogens on muscle is still in the exploratory phase, and the evidence that estrogen has a significant effect on muscle mass is lacking (31). Study have reported that estrogens have anti-catabolic effect on skeletal muscle, and hormone replacement therapy can preserve muscle mass (32). However, the effect of estrogen on skeletal muscle is not as well recognized as that of testosterone. HbA1c levels were found to be related to skeletal mass and to be an independent factor of sarcopenia in a multicenter cross-sectional study (33). Consistent with this study, the incidence of sarcopenia was higher in patients with diabetes, and diabetes was an independent contributor to sarcopenia in hemodialysis patients (34). However, the reason for the high incidence of sarcopenia in diabetic patients is not clear. One of the explanations is that insulin resistance is involved in skeletal muscle protein breakdown, and impaired insulin/IGF-I signaling could lead to a drop in phosphorylated Akt and muscle loss (35–37). Some studies reported that the level of insulin was inversely related to handgrip strength, and patients with insulin resistance had lower handgrip strength (38, 39). In addition to insulin, glucose may also be closely related to skeletal muscle maintenance, and hyperglycemia could inhibit muscle regeneration and accelerate sarcopenia (40). Serum magnesium was observed to be a protective factor against sarcopenia in our study. Magnesium is involved in the synthesis of protein and ATP and plays a key role in muscle metabolism and function (41, 42). A cross-sectional study including 396,283 participants also reported that higher serum magnesium was associated with lower odds of sarcopenia (43). Scott et al. investigated that magnesium supplementation was a positive predictor of change in muscle mass (β = 0.07, P=0.02), and it can ameliorate the progression of sarcopenia in older individuals (44). An observational study involving 156,575 patients and a cross-sectional study of 2570 women also showed that dietary magnesium is positively associated with skeletal muscle mass and grip strength (45, 46). Only one RCT including 139 healthy older women reported that 300 mg magnesium supplementation for 3 months would improve muscle mass (47). In the light of these studies, we expect more RCTs in the coming years to elucidate the effect of magnesium intake on sarcopenia.

It has been widely assumed that bone and skeletal muscle are interrelated tissues with shared mechanical and molecular mechanisms and are regulated by many common factors. Bone and muscle communicate in the “bone-muscle unit” through paracrine and endocrine signals to coordinate their development (48, 49). Bone metabolism disorder also accelerates the progression of sarcopenia in patients on MHD. The loss of muscle mass and function can also promote osteoporosis in reverse. In our study, skeletal muscle mass and strength were positively associated with BMD, while a correlation was not found between lumbar spine BMD and grip strength. This may be because aortic calcification is common in dialysis patients, which affects the measurement of lumbar spine BMD. Therefore, the lumbar spine is not a good site for bone density measurement in dialysis patients, especially those with aortic calcification. Our study showed that the rate of sarcopenia in patients with osteoporosis was 3.875 times than in those without osteoporosis. Yoshimura et al. found that patients with osteoporosis were 2.99 times more likely to develop sarcopenia than people without osteoporosis in four years (17). A systematic analysis of 38 studies including 224,321 participants suggested that sarcopenia increased the risk of osteoporosis by 3.06 times. In addition, this systematic analysis also included 7 trials involving 171,514 participants revealed that each standard deviation (SD) increase in relative appendicular skeletal muscle mass (RASM) was associated with a significant 35% reduction in osteoporosis risk (50). Ahn et al. reported that left hand grip strength was significantly associated with osteoporosis in female aged 60–69 years, while not found in aged 70 years and in the right hand (51).

Reduced muscle mass and strength, and low bone density are significantly associated with falls, fractures and mortality, which would contribute to a decline in the quality of life and an increase in the economic burden. Osteosarcopenia was proposed as a new concept to strengthen the awareness of healthy bone and muscle. At present, there are few studies on the epidemiology of osteosarcopenia, especially in hemodialysis patients. In our study, 22.3% of the patients presented with osteosarcopenia, higher than 5.8% in the general population and 17.2% in patients with kidney transplantation (52, 53). The age difference between osteosarcopenia and sarcopenia was smaller than that between osteoporosis patients, indicating that the progression from sarcopenia to osteosarcopenia is faster than that from osteoporosis. Muscle wasting could accelerate the loss of BMD; in terms of the pathological mechanism, mechanical contraction of muscles stimulates bone formation and prevents bone mineral loss (54, 55). Osteosarcopenia is a strong predictor of morbidity and mortality, as it could lead to lower quality of life and increased falls and fractures. A meta-analysis suggested that osteosarcopenia was significantly associated with the risk of mortality (OR 1.66, 95% CI 1.23–2.26), fracture (OR 2.46, 95% CI 1.83–3.30), and falls (OR 1.62, 95% CI 1.28–2.04) compared with nonosteosarcopenia (56). After adjusting for age, sex, diabetes, cardiovascular disease, and fracture history, osteosarcopenia remained an independent risk factor for all-cause mortality, while it disappeared in sarcopenia alone. Our study suggested that the coexistence of sarcopenia and osteoporosis increased the risk of all-cause death, meaning both bone and muscle are equally important. We expect more trials with large sample sizes to explore the prevalence of osteosarcopenia and its impact on fracture and death in hemodialysis patients.

Currently, scholars have a deep understanding of osteoporosis in dialysis patients, while the understanding and attention of sarcopenia, especially osteosarcopenia, are still insufficient. As a result, sarcopenia and osteoporosis tend to occur simultaneously for one person, and both are strongly associated with poor health outcomes. Therefore, attention should be given to muscle health as well as bone problems. Exercise and nutrition are critical to osteosarcopenia. The majority of studies have found that exercise may exert a beneficial effect on osteosarcopenia by improving muscle mass, strength and function, especially resistance training, which can increase the cross-sectional area and size of muscle fibers (57–59). A healthy diet plays an essential role in muscle and bone maintenance and preventing the progression of osteosarcopenia. It has been observed that proteins rich in leucine are more important in protein synthesis, and leucine supplementation increases anabolism and lean body mass (60, 61). For patients with dialysis, an increased amount of protein (1.0-1.2g/kg/day) is recommended (62). Vitamin D supplementation for sarcopenia remains controversial. Study has reported that 1,25-dihydroxyvitamin D can bind to vitamin D receptors on skeletal muscle to regulate the number and volume of type II muscle fibers and improve skeletal muscle strength and mass (63). A meta-analysis of 30 RCTs involving 5615 individuals showed that vitamin D supplementation had a small positive effect on muscle strength, but not found on muscle mass. And for people who presented low 25-hydroxyvitamin D level and aged 65 years or older, the effect on muscle strength was even more pronounced (64). While, a recent meta-analysis of 10 RCTs reported that vitamin D monotherapy did not improve any sarcopenia in ages >50 years old (65). We expect additional studies to explore it. Additionally, improving clinicians’ awareness of osteosarcopenia and testing muscle mass and grip strength along with bone mineral density examination will contribute to determining the presence of osteosarcopenia, taking effective intervention measures to prevent disease progression, and reducing the occurrence of poor outcomes.

Our study also had some limitations. First, our study was a single-center study with a small sample size; therefore, the study subjects cannot be generalized to all hemodialysis patients. Second, the results of the BIA test were affected by body water, and although the BIA test was performed after dialysis, it may still affect the measurement.

## Conclusion

Sarcopenia is highly present in MHD patients and is also consistently positively associated with osteoporosis. Osteosarcopenia is not rare and has a greater risk of mortality than either sarcopenia alone or osteoporosis alone. Early comprehensive evaluation and treatment of bone disorders and muscle mass and function loss is necessary. In addition, more clinical trials on the influence of osteosarcopenia and therapeutic interventions for muscle anabolism and bone disorders in dialysis patients are needed in the future.

## Data availability statement

The original contributions presented in the study are included in the article/supplementary material. Further inquiries can be directed to the corresponding authors.

## Ethics statement

The studies involving human participants were reviewed and approved by Ethics Committee of West China Hospital of Sichuan University, approval number (2020-446). The patients/participants provided their written informed consent to participate in this study.

## Author contributions

LZ conceived and designed the study. TX completed the literature searches and data analysis. TX drafted the manuscript. LZ and PF revised the final manuscript. All authors contributed to the article and approved the submitted version.
